# Hepatitis E Virus Infection in Patients With Chronic Liver Diseases: A Latin American Multicenter Study

**DOI:** 10.1093/infdis/jiaf615

**Published:** 2026-01-28

**Authors:** Anabella Clara Fantilli, María Belén Pisano, Maribel Martínez Wassaf, Guadalupe Di Cola, Domingo Balderramo, Pablo Romagnoli, Jhon Prieto, Marco Arrese, Enrique Carrera, Javier Díaz Ferrer, Angelo Z Mattos, Marina Fernández, Grisel Maribel Britos, María Eugenia Bernaschini, Santiago A Sepúlveda, Juan Carlos Roa, Sergio Grutadauria, Alina Zerega, Melina Ferreiro, Esteban González Ballerga, Jonathan Salmon, Andre Boonstra, José Daniel Debes, Viviana Elizabeth Ré

**Affiliations:** Facultad de Ciencias Médicas, Instituto de Virología “Dr. J.M. Vanella”, Universidad Nacional de Córdoba, Córdoba, Argentina; Consejo Nacional de Investigaciones Científicas y Técnicas (CONICET), Buenos Aires, Argentina; Facultad de Ciencias Médicas, Instituto de Virología “Dr. J.M. Vanella”, Universidad Nacional de Córdoba, Córdoba, Argentina; Consejo Nacional de Investigaciones Científicas y Técnicas (CONICET), Buenos Aires, Argentina; Departamento de Virología, LACE laboratorios, Córdoba, Argentina; Facultad de Ciencias Médicas, Instituto de Virología “Dr. J.M. Vanella”, Universidad Nacional de Córdoba, Córdoba, Argentina; Consejo Nacional de Investigaciones Científicas y Técnicas (CONICET), Buenos Aires, Argentina; Hospital Privado Universitario de Córdoba, Córdoba, Argentina; Consejo Nacional de Investigaciones Científicas y Técnicas (CONICET), Buenos Aires, Argentina; Hospital Privado Universitario de Córdoba, Córdoba, Argentina; Centro de Enfermedades Hepáticas y Digestivas (CEHYD), Bogotá, Colombia; Departamento de Gastroenterología, Facultad de Medicina, Pontificia Universidad Católica de Chile, Santiago, Chile; Departamento de Gastroenterología y Hepatología, Hospital Especialidades Eugenio Espejo, Universidad San Francisco de Quito, Quito, Ecuador; Universidad San Martin de Porres, Lima, Perú; Graduate Program in Medicine: Hepatology, Federal University of Health Sciences of Porto Alegre, Porto Alegre, Brazil; Hospital Privado Universitario de Córdoba, Córdoba, Argentina; Consejo Nacional de Investigaciones Científicas y Técnicas (CONICET), Buenos Aires, Argentina; Facultad de Matemática, Astronomía y Física, Universidad Nacional de Córdoba, Córdoba, Argentina; Consejo Nacional de Investigaciones Científicas y Técnicas (CONICET), Buenos Aires, Argentina; Facultad de Matemática, Astronomía y Física, Universidad Nacional de Córdoba, Córdoba, Argentina; Departamento de Anatomía Patológica, Facultad de Medicina, Pontificia Universidad Católica de Chile, Santiago, Chile; Departamento de Anatomía Patológica, Facultad de Medicina, Pontificia Universidad Católica de Chile, Santiago, Chile; Laboratorio Central, Sanatorio Allende, Córdoba, Argentina; Laboratorio Central, Sanatorio Allende, Córdoba, Argentina; División de Gastroenterología, Hospital de Clínicas José de San Martín (UBA), Buenos Aires, Argentina; División de Gastroenterología, Hospital de Clínicas José de San Martín (UBA), Buenos Aires, Argentina; División de Gastroenterología, Hospital de Clínicas José de San Martín (UBA), Buenos Aires, Argentina; Department of Gastroenterology and Hepatology, Erasmus MC, University Medical Center, Rotterdam, The Netherlands; Department of Gastroenterology and Hepatology, Erasmus MC, University Medical Center, Rotterdam, The Netherlands; Department of Medicine, University of Minnesota, Minneapolis, Minnesota, USA; Facultad de Ciencias Médicas, Instituto de Virología “Dr. J.M. Vanella”, Universidad Nacional de Córdoba, Córdoba, Argentina; Consejo Nacional de Investigaciones Científicas y Técnicas (CONICET), Buenos Aires, Argentina

**Keywords:** HEV seroprevalence, chronic liver disease, Latin America, alcohol-related liver disease, cirrhosis

## Abstract

**Background:**

Hepatitis E virus (HEV) is a major cause of acute hepatitis worldwide, yet its impact in Latin America remains underexplored. Evidence suggests that chronic liver disease (CLD) patients infected with HEV face increased risks of disease progression and mortality. The PROGINS haplotype has been proposed to influence susceptibility to HEV. This study assessed HEV infection in CLD patients from Latin America and potential associated factors, including the PROGINS haplotype.

**Methods:**

A total of 971 individuals—784 with CLD and 187 healthy controls (HC)—from six countries (Argentina, Brazil, Chile, Colombia, Ecuador, and Peru) were analyzed for anti-HEV IgG and IgM (ELISA), HEV-RNA (RT-qPCR and nested PCR with Sanger sequencing and phylogenetic analysis), and the PROGINS haplotype (PCR).

**Results:**

The overall anti-HEV IgG seroprevalence was 15.2%: 15.4% in CLD and 14.4% in HC, with no statistical difference. Marked geographical disparities were observed, with Chile showing the highest (45.1%) and Argentina the lowest (4.2%) anti-HEV IgG detection rates. Cirrhosis and alcohol-related liver disease (ALD) were significantly associated with higher detection rates, while neither age nor sex influenced HEV seroprevalences. PROGINS haplotype showed no significant association with HEV infection. Anti-HEV IgM and HEV-RNA were detected in 11.2% and 0.4% of participants, respectively. Phylogenetic analysis confirmed zoonotic HEV-3 circulation in the region.

**Conclusions:**

This first multinational assessment of HEV in Latin America reveals heterogeneous seroprevalence across countries. Findings support considering HEV testing in diagnostic protocols for CLD patients particularly those with cirrhosis or ALD- when presenting with unexplained hepatic decompensation or acute hepatitis.

## Teaser

This multicenter study across six Latin-American countries reveals heterogeneous hepatitis E seroprevalence in chronic liver disease patients, with higher rates in cirrhosis and alcohol-related liver disease, confirming zoonotic HEV-3 circulation and supporting HEV testing in patients with unexplained hepatic decompensation.

## Graphical Abstract

**Graphical Abstract:**
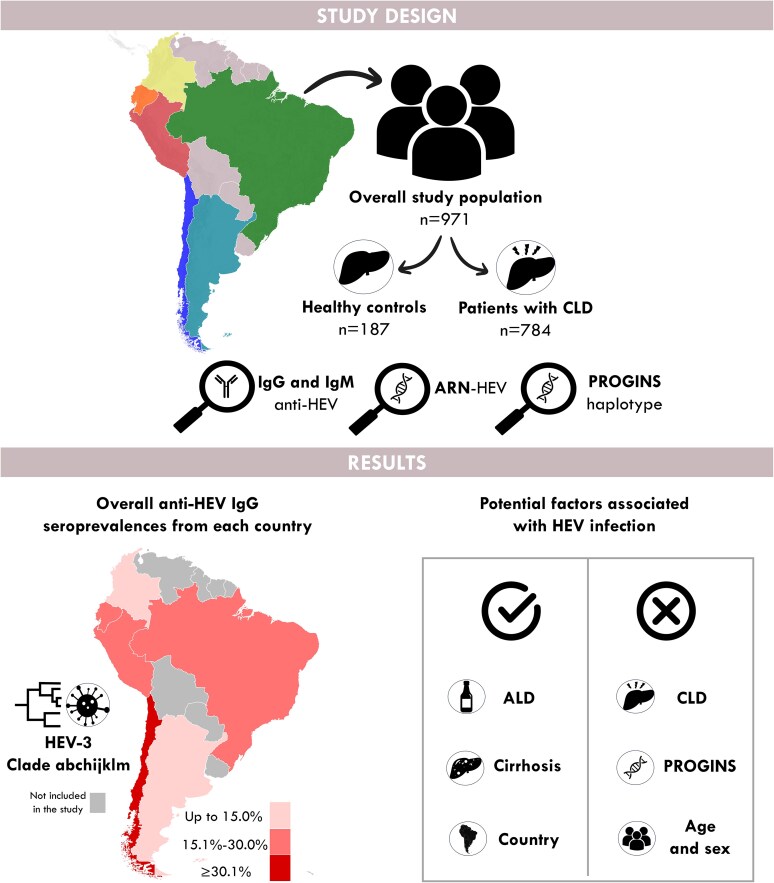
ALT TEXT Infographic showing a map of South America with highlighted countries and indicated sample sizes. Participants are categorized as healthy controls or patients with chronic liver disease. The figure illustrates the study methodology, including serological and molecular testing for anti-HEV IgG and IgM, HEV RNA detection, and analysis of the PROGINS haplotype. A heat map presents anti-HEV IgG seroprevalence by country. Factors assessed for their association with HEV infection include chronic liver diseases, cirrhosis, alcoholic liver disease, age, sex, and presence of the PROGINS haplotype.

The hepatitis E virus (HEV) is one of the main causes of acute hepatitis, with an estimated 20 million infections worldwide annually [1]. HEV (species *Paslahepevirus balayani*) is an RNA virus from the *Hepeviridae* family, divided into eight genotypes, of which genotypes 1 to 4 (HEV-1, HEV-2, HEV-3, and HEV-4) are most significant in human disease [2]. HEV-1 and HEV-2, transmitted through the fecal-oral route, circulate in endemic regions and cause large outbreaks, particularly in Asia, Africa, and Central America. In contrast, HEV-3 and HEV-4, primarily zoonotic, cause sporadic or clustered cases in resource-rich regions such as Europe, North America, and Japan [2]. Each genotype has been classified into subtypes and, for HEV-3, Smith et al. (2020) proposed two clades (abchijklm and efg) [3].

Most studies on HEV epidemiology originate from Europe, the Middle East, and North Africa [4]. In Latin America, HEV circulation has been reported mainly in Brazil and Argentina, with seroprevalence estimates ranging from 0% to 66.3%, depending on population group, country, and assay. Higher rates are observed among HIV-infected individuals, transplant recipients, patients on hemodialysis, and those with cirrhosis compared with healthy individuals [4, 5]. HEV-3 remains the most frequently detected genotype in human, animal, and environmental samples [5, 6]. Nevertheless, HEV remains underexplored in Latin America, where it is rarely included in national diagnostic algorithms and testing capacity is limited. This, combined with low clinical awareness, has likely led to substantial underreporting and limited understanding of HEV epidemiology in the region [5].

HEV-3 infections are often asymptomatic and subclinical, and the infection is primarily zoonotic and foodborne. Major risk factors include consumption of undercooked pork or game meat and contact with infected animals [2]. However, they pose significant risks to vulnerable populations, including immunocompromised individuals and patients with chronic liver diseases (CLD) [2]. In CLD patients, HEV infection can result in severe outcomes, including acute-on-chronic liver failure (ACLF) or progression to chronic hepatitis E. Additionally, HEV infection may accelerate pre-existing liver conditions and increase mortality risk [2, 7–9]. Only four publications from Latin America, from Argentina and Brazil, have reported HEV seroprevalences among CLD patients [10–13]. Moreover, two HEV-related-ACLF cases have been reported: one in Peru [14] and one in Argentina [15].

Several host factors, including immunosuppression, pregnancy, and genetic predisposition, have been identified as determinants of HEV infection severity and mortality [2, 16]. Among them genetic variations in the progesterone receptor (PR) gene stand out [16]. PROGINS haplotype -consisting of a 320-bp insertion in intron G and point mutations in exons 4 and 5- is associated with reduced transcript levels and lower progesterone response, altering its immune-regulatory effects [16]. PROGINS has been linked to increased HEV susceptibility [17], and higher risk of acute hepatitis and liver failure in HEV-infected pregnant women [16] and immunocompromised individuals, such as HIV-positive and liver transplant recipients, with controversial results [17–19]. Currently no data exist on PROGINS presence in CLD patients and its possible link to HEV infection.

This study used a large sample databank from six Latin American countries through the ESCALON project (H2020/825510) to investigate HEV infection in CLD patients and identify potential associated factors, including the PROGINS haplotype.

## METHODS

### Samples

We analyzed 971 patients from Latin America. Among these, 784 samples were from individuals with CLD, recruited from six South American countries as part of the ESCALON consortium (www.escalon.eu) between 2019 and 2023 in major hospitals: Argentina (n = 224 from Cordoba and Buenos Aires), Brazil (n = 69 from Porto Alegre), Chile (n = 115 from Santiago), Colombia (n = 259 from Bogotá), Ecuador (n = 61 from Quito), and Peru (n = 56 from Lima). Additionally, 187 samples from healthy controls (HC) were collected in Argentina, Brazil, Chile, and Colombia.

The sample size of individuals from each country for all participant groups including CLD and HC is described in Supplementary Table 1.

Briefly, recruitment of patients and HC for the ESCALON project was based on sample availability. Individuals aged 18 years or older with CLD of various etiologies were included, comprising metabolic dysfunction–associated steatotic liver disease (MASLD) (n = 406), alcohol-related liver disease (ALD) (n = 157), viral hepatitis (chronic hepatitis B and/or C) (n = 143), and other etiologies (n = 128). Some patients presented multiple etiologies and were also classified as having hepatocellular carcinoma (HCC) and/or cirrhosis (Supplementary Table 2). The ESCALON diagnostic criteria are described in Goble et al., 2023 [20]. HC, defined as individuals without CLD, were matched to the CLD cohort by age distribution.

Clinical information regarding age, sex, CLD etiology and comorbidities was obtained from the ESCALON REDCap database [21, 22].

Pre-existing comorbidities included diabetes (n = 261), hypertension (n = 287), renal disease (n = 23), dyslipidaemia (n = 120), coronary disease (n = 42), and others (n = 451).

### Serological Tests

Anti-HEV IgG and IgM antibodies were tested by third-generation ELISA (Diapro, Italy; specificity ≥98%, sensitivity ≥98%). Results were based on the sample (S)/cut-off (CO) ratio: < 0.9 negative, 0.9–1.1 equivocal, > 1.1 positive. All samples were tested for anti-HEV IgG, and IgM was assessed in positive or equivocal samples.

### HEV Molecular Detection, Genotyping, and Phylogenetic Analysis

A subset of samples (n = 561), stored under conditions suitable for molecular biology analyses and with sufficient volume for nucleic acid extraction, were tested for HEV RNA regardless of serological status. RNA/DNA Mini Kit (Thermo Fisher). HEV RNA detection targeted ORF-3 (70 bp) via real-time RT-PCR (TaqMan® Fast, Applied Biosystems) [23]. RNA-positive samples were further analyzed by RT-nested PCR for ORF-2 (348 bp) and ORF-1 (287 bp). cDNA synthesis used ImProm-II reverse transcriptase (Promega), and PCR products were sequenced by Sanger on a Genetic Analyzer 3500×L (Applied Biosystems).

Phylogenetic analysis used 750 ORF-2 sequences (342 bp), including this study's isolate (GenBank PQ310681) and HEV-3 clade abchijklm sequences available until January 2024. Analyses were performed in IQ-TREE v2.1 [24] using maximum likelihood with SH-aLRT (1000) and ultrafast bootstrap (10 000) for robustness.

### PROGINS Detection in CLD Patients

Genomic DNA was extracted from whole blood. Among anti-HEV IgG-positive CLD patients, 98 samples were analyzed for PROGINS haplotype, and IgG-negative CLD patients (n = 119) were also included, depending on whole blood sample availability.

DNA was isolated from 2 mL whole blood using the Gentra Puregene kit (Qiagen). PCR with specific primers detected PROGINS haplotype [25], identifying homozygous wild type (WT), homozygous mutant, or heterozygous. Patients were grouped as PROGINS carriers (homozygous and heterozygous) or non-carriers (homozygous WT).

### Statistical Analyses

Proportions with 95% confidence intervals (CIs) were calculated for dichotomous variables. Median and interquartile ranges (IQR) were reported for age. Recent HEV infection was defined by positive IgM and/or RNA.

Seroprevalence across groups was assessed using generalized linear mixed models (GLMM) with binomial responses and logit link functions. Geographical regions were included as random effects, while age and sex were fixed effects. Comorbidity was excluded, as it did not improve model fit, assessed by Likelihood Ratio Test (LRT). Categorical variable significance was also tested via LRT.

Log-linear models assessed seroprevalence across countries; Fisher's Exact Test compared IgG seroprevalence by CLD/HC status. Holm-adjusted *P*-values were used for multiple comparisons. Association with PROGINS was analyzed with GLMM and Fisher's test.

Odds ratios with 95% CIs were reported. *P*-values <.05 were considered significant. Analyses were conducted in R.

### Ethics Statement

The study was conducted according to the guidelines of the Declaration of Helsinki (1964, amended 2008), and approved by the Ethics Committee RePIS-3817, Ministry of Health of Córdoba, Argentina, and each participating center obtained local ethical approval. Written informed consent was obtained from all participants enrolled in the ESCALON project, which included the investigation of hepatotropic viruses as potential risk factors for CLD.

## RESULTS

### Demographic and Baseline Characteristics of the Study Cohort

A total of 971 individuals from six Latin American countries were included. The main characteristics are summarized in Table 1. Of the 784 CLD patients, 627 (80.0%) had cirrhosis and 215 (27.4%) HCC. Baseline information by country is detailed in Supplementary Table 2.

**Table 1.** Baseline Characteristics and Anti-HEV IgG Seroprevalence of Patients With CLD and HC

Cohort	N (%)	Age (median, IQR)	Sex (n, % Male)	Anti-HEV IgG + (n, %) [95% CI]
**Overall**	971 (100.0)	63.0, 14.0	458/971, 47.2	148/971, 15.2 [13.0–17.6]
**CLD**	784/971 (80.7)	63.0, 12.2	425/784, 54.2	121/784, 15.4 [13.0–18.1]
Cirrhosis	627/784 (80.0)	64.0, 12.0	359/627, 57.3	117/627, 18.7 [15.7–21.9]
HCC^a^	215/784 (27.4)	67.0, 11.0	139/215, 64.7	33/215, 15.3 [10.8–20.9]
**HC**	187/971 (19.3)	61.4, 20.5	33/187, 17.6	27/187, 14.4 [9.7–20.3]

Abbreviations: 95% CI, 95% confidence interval for proportions; CLD, chronic liver disease; HC, healthy controls; HCC, hepatocellular carcinoma; IQR, interquartile range.

^a^89.8% (193/215) of HCC patients also had cirrhosis.

### Anti-HEV IgG Seroprevalences Across Latin American Countries

**Figure 1.**
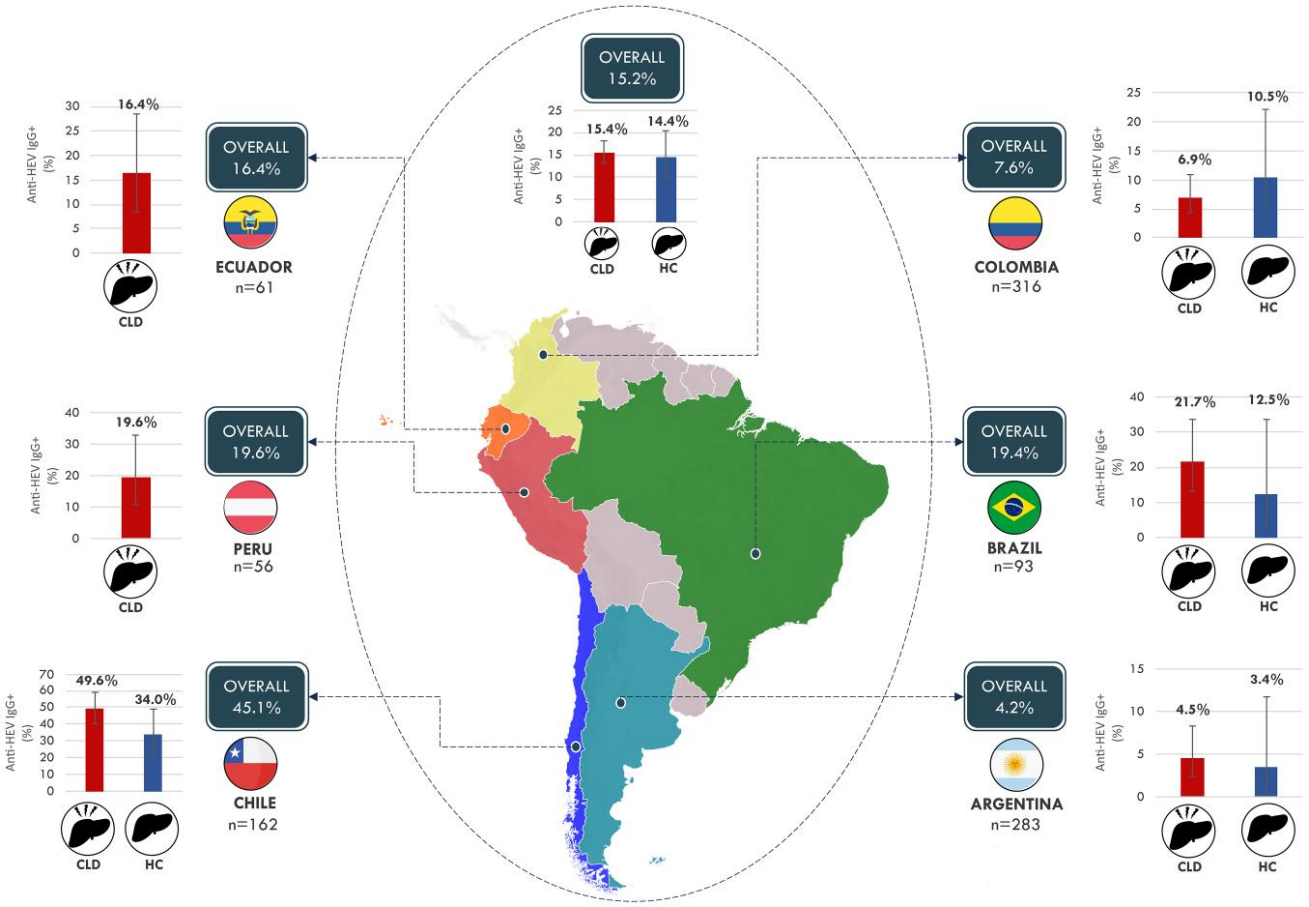
Overall anti-HEV IgG seroprevalences in HC and CLD patients from each country. Numbers above bars indicate specific seroprevalence for each group; error bars indicate 95% Confidence Intervals. Fisher's Exact Test was used to compare anti-HEV IgG seroprevalence between CLD patients and healthy controls, but no statistically significant differences were observed. CLD, chronic liver diseases; HC, healthy controls.

The overall anti-HEV IgG seroprevalence was 15.2%: 15.4% in CLD and 14.4% in HC, with no statistical differences (Table 1). When stratified by country, the highest seroprevalence was observed in Chile at 45.1% (73/162, 95% CI 37.2–53.1), and the lowest in Argentina (4.2%, 12/283, 95% CI 2.7–8.2). Figure 1 shows seroprevalences in HC and CLD by country.

Higher IgG rates were found in CLD compared with HC in all countries with both groups, except Colombia, where HC exceeded CLD. However, differences were not statistically significant (Figure 1).

Regional variations were significant: odds of being IgG-positive were higher in Chile than all others (*P* < .001). Argentina had significantly lower odds than other countries except Colombia. This pattern was consistent overall and within CLD and HC groups.

### Association of Clinical and Demographic Variables With Anti-HEV IgG Seroprevalence

**Figure 2.**
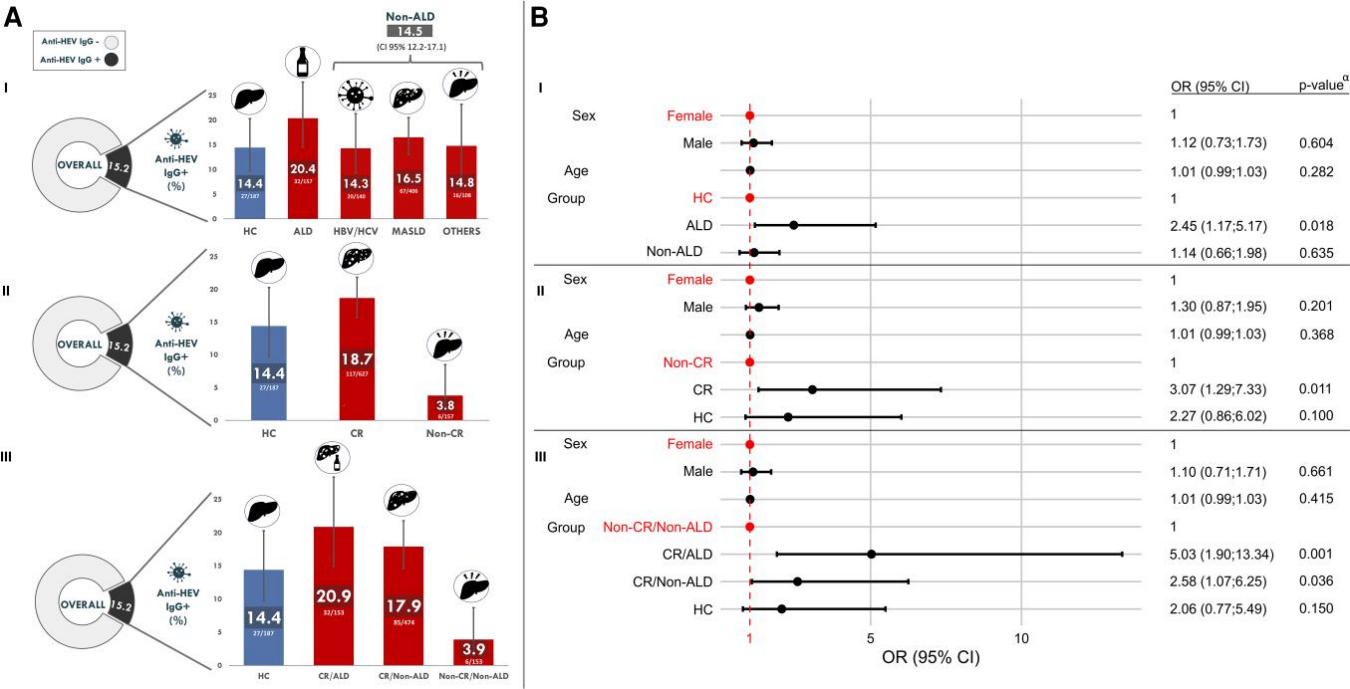
(*A*): Overall anti-HEV IgG seroprevalence in the total study population (15.2%) and stratified by different categories: (I) CLD etiologies and HC individuals; (II) individuals with cirrhosis (CR) and without cirrhosis (non-CR) and HC group; and (III) individuals with and without CR alongside ALD etiology and HC individuals (Non-CR/ALD group was not included due to low sample size). Numbers above bars indicate specific seroprevalence for each group; error bars indicate 95% CIs. (*B*): Odds ratios derived from binomial GLMMs, with 95% CIs and *P*-values. HC, healthy controls; ALD, alcoholic liver disease; non-ALD, patients without alcoholic liver disease; MASLD, metabolic dysfunction–associated steatotic liver disease; CR, cirrhosis; non-CR, patients without chirrosis; CR/ALD, patients with cirrhosis and alcoholic liver disease; CR/Non-ALD, patients with cirrhosis and without alcoholic liver disease; Non-CR/Non-ALD, patients without chirrosis or alcoholic liver disease; 95% CI, 95% confidence interval for proportions; OR, odds ratios; GLMM (generalized linear mixed model). ^α^P-values were obtained from LRT tests applied within binomial GLMMs with logit link functions. *P*-values <.05 were considered significant.

Individuals with cirrhosis showed higher IgG rates (18.7%; 117/627) than those without (3.8%; 6/157). Odds of being IgG + were 3.07 times higher in patients with cirrhosis compared with patients with CLD without cirrhosis (*P* = .01) (Figure 2; Supplementary Table 3). Considering cirrhosis and its etiology simultaneously significantly affected HEV seroprevalence (LRT X^2^ = 13.2, *P* = .004; Supplementary Table 4). Anti-HEV IgG detection rates were higher in alcohol-related cirrhosis patients (20.9%, OR 5.03) than in individuals with non-cirrhotic CLD of other etiologies (3.9%, *P* = .001) (Figure 2). Subgroup analysis considering only CLD patients, showed cirrhosis (LRT X²=4.2, *P* = .039) and ALD (LRT X^²^=4.5, *P* = .033) significantly influenced IgG seroprevalence (Table 2).

**Table 2.** Analysis of Deviance of the Binomial Generalized Linear Mixed Models for the Effect of Sex, Age, the Presence of ALD and Cirrhosis as Fixed Effects, and Geographical Region as Random Effect, on Anti-HEV IgG Seroprevalences Among CLD Patients

Variable	AIC	LRT X^2^	*P* Value^a^
Age	569.1	2.3	.129
Sex	567.9	1.0	.305
ALD	571.4	4.5	.033 *
Cirrhosis	571.1	4.2	.039 *

Abbreviations: AIC, Akaike information criterion; ALD, alcoholic liver disease; LRT, Likelihood Ratio Test.

^a^ *P*-values were obtained from LRT tests applied within binomial GLMMs with logit link functions. * *P*-values <.05 were considered significant.

When CLD patients were reclassified by ALD status, ALD significantly influenced HEV seroprevalence (LRT X² = 7.4, *P* = .024; Supplementary Table 5). ALD patients had higher rates (20.4%) than HC (14.4%) and non-ALD (14.5%) (Figure 2). Pairwise comparisons showed ALD patients had 2.45 (*P* = .018) and 2.1 (*P* = .022) times higher odds of being IgG + than HC and non-ALD, respectively (Supplementary Table 5).

Overall, GLMMs consistently identified ALD and cirrhosis as factors associated with a higher risk of HEV infection, while age and sex had no significant effects.

HCC patients had anti-HEV IgG seroprevalence of 15.3% (Table 1), with no significant differences compared with patients with cirrhosis or HC.

### Recent HEV Infections

Overall anti-HEV IgM positivity was 11.2% (20/179, 95% CI 7.1–16.9). All these individuals were also anti-HEV IgG+. Mean age was 63 (IQR 9), 60.0% male, and 55.0% were from Chile. IgM positivity was 9.4% (14/149, 95% CI 5.4–15.6) in CLD patients—78.6% male, 42.8% from Chile, median age 60- and 20.0% (6/30, 95% CI 8.4–39.1) in HC −16.7% male, 66.7% from Chile, median age 61 (Supplementary Table 6).

Of 561 patients analyzed by real-time-RT-PCR, two were RNA-HEV positive (0.4%, 95% CI .0–1.4), both males from Buenos Aires, Argentina, with cirrhosis and HCC, negative for IgG and IgM. One was coinfected with HCV and had ALD, the other had hemochromatosis. Both presented jaundice, high bilirubin levels and normal transaminases (Supplementary Table 6). One was RT-nested PCR positive and sequenced.

### Phylogenetic Analysis

**Figure 3.**
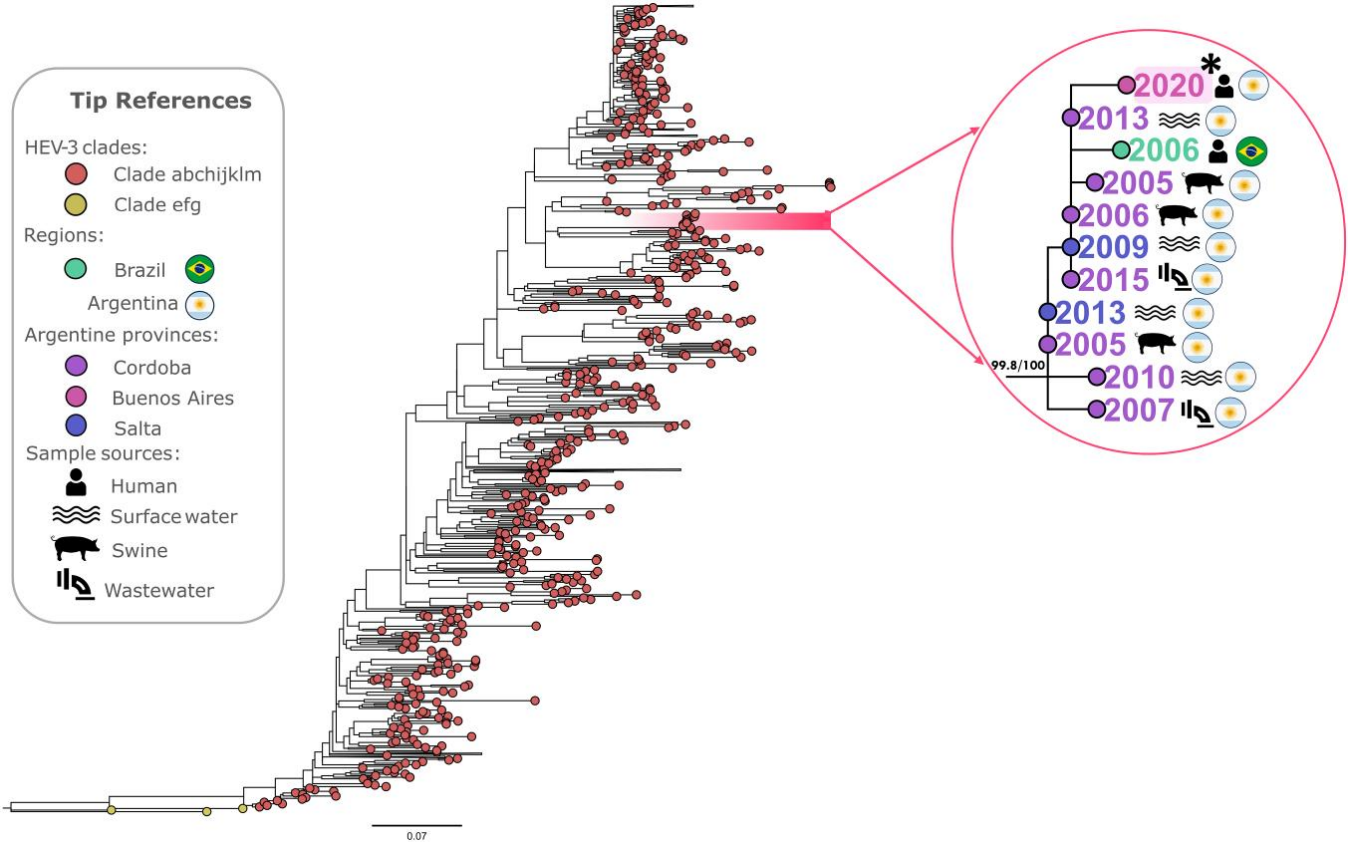
Maximum likelihood phylogenetic tree based on ORF-2 genomic region (length = 342 bp) of HEV-3 (reference sequences from clades abchijklm in red and efg in yellow). The monophyletic group that includes the sequence isolated from the sample of this study (highlighted and indicated with a *), is shown with more detail (99.8% SH-aLRT and 100.0% UFB supports for the group).

The isolated sample belonged to HEV-3, clade abchijklm, and grouped with high branch support (99.8% SH-aLRT/100.0% UFB) with sequences from Brazil and provinces of Argentina (Buenos Aires, Salta, and Cordoba), from various years and matrices (swine, wastewater, clinical samples, and recreational water) (Figure 3).

### Association Between PROGINS Status and HEV Infection

Of the 98 IgG-positive patients, 20.4% were PROGINS carriers. In comparison, 25.2% of the IgG-negative patients were PROGINS carriers. Baseline characteristics of patients, categorized by PROGINS status and HEV serology, are summarized in Table 3.

**Table 3.** Baseline Characteristics of Patients Analyzed by PROGINS and HEV Status

Characteristic	Anti-HEV IgG + (n, %) [95% CI]	Anti-HEV IgG− (n, %) [95% CI]	TOTAL (n, %) [95% CI]
**PROGINS carriers**	20/98, 20.4 [13.2–30.0]	30/119, 25.2 [17.9–34.2]	50/217, 23.0 [17.7–29.3]
Age (median, IQR)	64.0, 8.5	62.0, 14.0	62.5, 13.7
Sex (n, % male)	9/20, 45.0	15/30, 50.0	24/50, 48.0
**Non-PROGINS carriers**	78/98, 79.6 [70.0–86.8]	89/119, 74.8 [65.8–82.1]	167/217, 76.9 [70.7–82.3]
Age (median, IQR)	64.0, 11.0	64.0, 11.0	64.0, 12.0
Sex (n, % male)	50/78, 64.1	41/89, 46.1	91/167, 54.5
**TOTAL**	98	119	217
Age (median, IQR)	64.0, 11.0	63.0, 13.5	63.0, 12.0
Sex (n, % male)	59/98, 60.2	56/119, 47.1	115/217, 53.0

Abbreviations: 95% CI, 95% confidence interval for proportions; IQR, interquartile range.

The GLMM showed no association between the PROGINS haplotype and HEV infection in this cohort. When analyzing the association between the PROGINS haplotype and HEV infection within each country individually, no statistically significant association was observed (data not shown).

## DISCUSSION

This multicenter study examined anti-HEV IgG prevalence across several Latin American countries, analyzing healthy controls and individuals with CLD of various etiologies. We used the same seroassay for anti-HEV IgG and IgM detection, ensuring uniform test results across cohorts and offering insights into HEV circulation where data have been scarce. Moreover, this study provides details on the molecular epidemiology of the virus and genetic analysis of potential predispositions. To our knowledge, it represents the first report among CLD patients in Chile, Colombia, and Peru, the first documented HEV epidemiology from Ecuador, and the first comprehensive study from Latin America.

In our study, CLD presence was not significantly associated with higher anti-HEV IgG seropositivity, either overall or by country. This contrasts with reports linking CLD to impaired immune function and greater vulnerability to other hepatitis infections, including HEV [7]. Studies in China, Vietnam, Nepal, France, the UK, Taiwan, and the United States have shown higher HEV seroprevalence and mortality among CLD patients compared with the general population [7, 8, 26, 27].

Similarly, studies from Cameroon and China reported higher anti-HEV IgG prevalence among HCC patients than in those with CLD without HCC or healthy controls [28, 29]. In contrast, our results did not show a significant association between HCC and HEV seroprevalence, consistent with reports that also failed to demonstrate a clear link [30, 31]. Notably, nearly 90% of HCC patients in our study also had cirrhosis, making it difficult to assess whether HCC itself is independently associated with HEV infection.

The absence of a marked difference in HEV seroprevalence between CLD patients—including those with HCC—and HC should be interpreted with caution, as it may reflect factors such as variability in immune response, cohort heterogeneity, and sample size limitations.

Significant disparities in seroprevalence were observed among countries, with Chile exhibiting substantially higher odds of anti-HEV IgG positivity. Previous studies on anti-HEV IgG seroprevalence in Latin America over the past decade, focused on general populations and blood donors, have reported wide ranges. In Brazil, the most studied country in the region, rates differ widely between the North (0.9%) and South (65.5%) [6]. Argentina has reported lower rates, from 4% to 16.7% in the general population [5]. Limited data exist for Colombia, Chile, and Peru, with HEV seroprevalence in blood donors reaching 30.1% in Chile [32] and 45.2% in Colombia's Antioquia region [33]. Peru has recently reported 14.3% in general population [34], and for Ecuador, no prior data were available. Remarkably, the highest and lowest seroprevalence values were observed in neighboring countries, Chile and Argentina. While age and sex were not significantly associated with HEV positivity, the contrast may reflect regional dietary and cultural practices. In particular, as a coastal country, Chile likely has higher consumption of seafood, which has been reported as a potential transmission route for HEV [2], and combined with differences in food preparation, water sources, and potential exposure to livestock, these factors may help explain the elevated anti-HEV IgG seroprevalence compared with Argentina.

It cannot be excluded that these regional variations in published studies are methodological, attributed to differences in the sensitivity and specificity of the serological kits used [5, 6], since the lack of standardization in commercial assays complicates comparisons and interpretations of HEV seroepidemiological studies [35] However, a strength of our study is the consistent use of one serological method, ensuring accurate estimates and comparable prevalence data across Latin American countries.

Our findings reveal significantly higher HEV IgG rates in the cirrhosis group (18.7%) compared with those without cirrhosis (3.8%). Multiple studies from Europe and Asia support our results, identifying cirrhosis of different etiologies as a risk factor for higher HEV infection rates [7–9, 27, 36–42]. Particularly relevant for Latin America, our team also reported HEV seroprevalence of 25% among subjects with cirrhosis versus 4% in healthy controls in Argentina [10]. In Brazil, HEV seroprevalence in HCV patients ranged from 10.2% to 12% [11, 12], with higher rates in those with cirrhosis [11], although these are similar to rates in blood donors and the general population [6, 13], reflecting ongoing controversies.

Although our findings indicate an association between cirrhosis and higher anti-HEV IgG seroprevalence, the direction of causality remains uncertain. Some authors suggest that cirrhosis represents an immunocompromised state [9, 38]. Abundant evidence supports the presence of cirrhosis-associated immune dysfunction (CAID), marked by defects in innate and adaptive immunity that increase susceptibility to bacterial and viral infections [43]. Conversely, the significantly higher seroprevalence observed in cirrhotic patients compared with those with non-cirrhotic CLD may point to a potential role of HEV in accelerating the progression from chronic hepatitis to cirrhosis. HEV—typically self-limiting in immunocompetent hosts—may persist or precipitate acute hepatic decompensation in CLD patients, potentially contributing to disease progression or causing ACLF with high mortality [9]. Further studies are warranted to elucidate this relationship, as emphasized by the European Association for the Study of the Liver (EASL) recommendations to test for HEV in patients with unexplained flares of CLD [44].

We found no significant differences in anti-HEV IgG positivity rates across CLD etiologies, except for ALD, which was associated with higher HEV infection rates. Interaction between HEV and excessive alcohol consumption has been widely studied, identifying alcohol as a risk factor for the clinical manifestation of HEV infection [31, 45]. Higher anti-HEV IgG seroprevalence has also been reported in alcohol-related cirrhosis compared with other causes [27, 41]. Similarly, our group previously found higher HEV seropositivity in alcohol-related cirrhosis (39.5%) than in other etiologies (12.4%) [10]. We documented a case in Argentina of a patient with alcohol-related cirrhosis who developed ACLF after HEV-3 infection [15]. The precise mechanism remains unclear. It has been proposed that alcohol may increase viral replication, weaken immune responses, and enhance oxidative stress [46]. Notably, binge drinking induces bacterial translocation into the bloodstream, potentially making individuals more vulnerable to viral translocations as well [47].

In this study, we examined for the first time the progesterone receptor haplotype (PROGINS) and its association with HEV in CLD in Latin America. Only three previous studies evaluated PROGINS in non-pregnant immunosuppressed cohorts: two by Debes et al. suggested an association with HEV seropositivity in liver transplant recipients and HIV + patients [17, 18], and López-López et al. reported a protective role against HEV in HIV-infected women [19]. Our results did not find any association, suggesting the mutation is unlikely a risk factor for HEV in this setting.

We detected HEV-RNA in only two serum samples (0.4%). Consistent with our results, several studies reported low HEV-RNA rates in CLD patients due to a brief transient viraemia during acute infection [7, 8, 13, 38, 40, 45]. Notably, both HEV-RNA-positive cases were negative for anti-HEV IgM and IgG, which represents an unexpected finding compared with previous studies reporting that IgM-negative samples are generally RNA-negative, such as the large study conducted in Bangladesh [48]. The higher IgM positivity observed among healthy controls compared with CLD patients may be influenced by the small number of IgM-positive cases and by differences in sex distribution and geographic origin between groups. It is also worth noting that anti-HEV IgM rates were obtained only among individuals who were anti-HEV IgG positive. These factors, along with the small number of RNA-positive, limit broader conclusions about recent HEV infection in this cohort.

The sample that could be sequenced was identified as HEV-3 within clade abchijklm, consistent with prior findings in Argentina and South America [4–6]. Phylogenetic analyses revealed that the sample grouped in the same monophyletic cluster with 99.8/100% of support alongside sequences from surface water, wastewater, swine, and human sources from Argentina and Brazil across multiple years, indicating a close genetic relationship and revealing persistent HEV infections with the same strains among human and swine populations throughout time within the country [49, 50]. These analyses provide further evidence that, similar to European countries where HEV-3 predominates, HEV infections of zoonotic origin might play a role in South America.

This study has some methodological limitations. Primarily, there were no available epidemiological data on certain risk factors previously linked to HEV infection, such as recent animal contact, diet, blood transfusions, and high-risk occupations, leaving some questions unanswered. Secondarily, the heterogeneity of the sample—with considerable diversity in patient categories, liver disease etiologies, and stages—made statistical analysis challenging, particularly for subgroup analyses that require sufficient power to detect effects, which may restrict generalizability. Additionally, the healthy control group showed a markedly different sex distribution compared with the other groups. Finally, another limitation is potential sampling bias, as all collection sites were major hospitals within each city. However, the large number of samples from diverse geocultural backgrounds provides unique and novel information from an underrepresented region in HEV research.

Our study adds information on HEV circulation among healthy control cohorts and CLD patients across Latin America. Particularly in Chile, HEV may be highly endemic, underscoring the need for increased screening efforts and awareness.

In light of the significantly higher rates of HEV infection among individuals with ALD and cirrhosis -and given that more severe courses of HEV infection have previously been reported in these populations- HEV testing should be considered during the initial diagnostic workup in these patients, particularly when they develop acute liver dysfunction or unexplained liver decompensation, to enhance early detection of HEV infections. Additionally, preventive strategies should prioritize high-risk groups, such as promoting sanitation practices and the avoidance of undercooked meat (pork, wild boar and venison) and shellﬁsh, as recommended by the EASL guidelines. Further research is needed to better identify high-risk groups within the CLD population and assess whether HEV screening in specific cohorts could improve disease management and patient outcomes.

## Supplementary Material

jiaf615_Supplementary_Data

## Supplementary Data

Supplementary materials are available at *The Journal of Infectious Diseases* online (http://jid.oxfordjournals.org/). Supplementary materials consist of data provided by the author that are published to benefit the reader. The posted materials are not copyedited. The contents of all supplementary data are the sole responsibility of the authors. Questions or messages regarding errors should be addressed to the author.

## References

[1] World Health Organization . Hepatitis E—Fact Sheet. https://www.who.int/news-room/fact-sheets/detail/hepatitis-e. Accessed 15 October 2025.

[2] Kamar N, Izopet J, Pavio N, et al Hepatitis E virus infection. Nat Rev 2017; 3:17086. 10.1038/nrdp.2017.86 29154369

[3] Smith DB, Izopet J, Nicot F, et al Update: proposed reference sequences for subtypes of hepatitis E virus (species Orthohepevirus A). J General Virol 2020; 101:692–8. 10.1099/jgv.0.001435 PMC7660235 32469300

[4] Villalobos NVF, Kessel B, Rodiah I, Ott JJ, Lange B, Krause G. Seroprevalence of hepatitis E virus infection in the Americas: estimates from a systematic review and meta-analysis. PLoS One 2022; 17:e0269253. 35648773 10.1371/journal.pone.0269253 PMC9159553

[5] Pisano MB, Mirazo S, Re VE. Hepatitis E virus infection: is it really a problem in Latin America? Clin Liver Dis (Hoboken) 2020; 16:108–13. 33005391 10.1002/cld.931 PMC7508784

[6] de Oliveira JM, dos Santos DRL, Pinto MA. Hepatitis E virus research in Brazil: looking back and forwards. Viruses 2023; 15:548. 36851763 10.3390/v15020548 PMC9965705

[7] Qiu LX, Huang Y, Quan JL, et al Prognosis of hepatitis E infection in patients with chronic liver disease: a meta-analysis. J Viral Hepat 2023; 30:101–7. 36177994 10.1111/jvh.13754

[8] Hoan NX, Van Tong H, Hecht N, et al Hepatitis E virus superinfection and clinical progression in hepatitis B patients. EBioMedicine 2015; 2:2080–6. 26844288 10.1016/j.ebiom.2015.11.020 PMC4703726

[9] Yang H, Wen J, Zhang Q, et al Clinical characteristics of 1279 patients with hepatitis e in Tianjin. Epidemiol Infect 2023; 151:e157. 37704376 10.1017/S0950268823001516 PMC10548536

[10] Fantilli AC, Trinks J, Marciano S, et al Unexpected high seroprevalence of hepatitis e virus in patients with alcohol-related cirrhosis. PLoS One 2019; 14:1–9. 10.1371/journal.pone.0224404 PMC6812777 31648288

[11] Bricks G, Senise JF, Pott H Jr, et al Previous hepatitis E virus infection, cirrhosis and insulin resistance in patients with chronic hepatitis C. Br J Infect Dis 2019; 23:45–52. 10.1016/j.bjid.2019.02.002 PMC9428018 30836071

[12] Zitelli PMY, Gomes-Gouvêa M, Mazo DF, et al Hepatitis E virus infection increases the risk of diabetes and severity of liver disease in patients with chronic hepatitis C virus infection. Clinics 2021; 76:e3270. 34852140 10.6061/clinics/2021/e3270 PMC8595601

[13] Costa MB, Gouvêa MSG, Chuffi S, et al Seroprevalence of hepatitis E virus in risk populations and blood donors in a referral hospital in the south of Brazil. Sci Rep 2021; 11:6011. 33727656 10.1038/s41598-021-85365-5 PMC7966736

[14] Valenzuela V, Pinto J, Padilla M, et al Severa descompensación por virus de hepatitis E en una paciente con hepatitis autoinmune: reporte de un caso. Rev gastroenterol Perú 2012; 32:187–91. 23023183

[15] Fantilli A, López Villa SD, Zerega A, et al Hepatitis E virus infection in a patient with alcohol related chronic liver disease: a case report of acute-on-chronic liver failure. Virol J 2021; 18:1–6. 34886883 10.1186/s12985-021-01714-w PMC8662871

[16] Bose PD, Das BC, Kumar A, Gondal R, Kumar D, Kar P. High viral load and deregulation of the progesterone receptor signaling pathway: association with hepatitis E-related poor pregnancy outcome. J Hepatol 2011; 54:1107–13. 21145845 10.1016/j.jhep.2010.08.037

[17] Debes JD, Groothuismink ZMA, de Man RA, Boonstra A. Association between a progesterone receptor mutation and hepatitis E sero-positivity in liver transplant recipients. J Med Virol 2020; 92:3871–4. 32603532 10.1002/jmv.26236 PMC7772260

[18] Debes JD, Pas SD, Groothuismink ZMA, van der Ende ME, de Man RA, Boonstra A. A mutation in the progesterone receptor predisposes to HEV infection in HIV-positive patients. Liver International 2018; 38:792–6. 29285885 10.1111/liv.13678 PMC5947588

[19] López-López P, Rivero-Juarez A, Frias M, et al Mutations in the progesterone receptor (PROGINS) may reduce the symptoms of acute hepatitis E and protect against infection. Front Microbiol 2019; 10:1–6. 31787965 10.3389/fmicb.2019.02617 PMC6854998

[20] Goble S, Akambase J, Prieto J, et al MBOAT7 rs641738 variant is not associated with an increased risk of hepatocellular carcinoma in a Latin American cohort. Dig Dis Sci 2023; 68:4212–20. 37684433 10.1007/s10620-023-08104-y PMC10570183

[21] Harris PA, Taylor R, Minor BL, et al The REDCap consortium: building an international community of software platform partners. J Biomed Inform 2019; 95:103208. 31078660 10.1016/j.jbi.2019.103208 PMC7254481

[22] Harris PA, Taylor R, Thielke R, Payne J, Gonzalez N, Conde JG. Research electronic data capture (REDCap)—a metadata-driven methodology and workflow process for providing translational research informatics support. J Biomed Inform 2009; 42:377–81. 18929686 10.1016/j.jbi.2008.08.010 PMC2700030

[23] Jothikumar N, Cromeans TL, Robertson BH, Meng XJ, Hill VR. A broadly reactive one-step real-time RT-PCR assay for rapid and sensitive detection of hepatitis E virus. J Virol Methods 2006; 131:65–71. 16125257 10.1016/j.jviromet.2005.07.004

[24] Minh BQ, Schmidt HA, Chernomor O, et al IQ-TREE 2: new models and efficient methods for phylogenetic inference in the genomic era. Mol Biol Evol 2020; 37:1530–4. 32011700 10.1093/molbev/msaa015 PMC7182206

[25] Agoulnik IU, Tong X-W, Fischer D-C, et al A germline variation in the progesterone receptor gene increases transcriptional activity and may modify ovarian cancer risk. J Clin Endocrinol Metab 2004; 89:6340–7. 15579801 10.1210/jc.2004-0114

[26] Wong RJ, Cheung R, Gish RG, Chitnis AS. Prevalence of hepatitis E infection among adults with concurrent chronic liver disease. J Viral Hepat 2021; 28:1643–55. 34415657 10.1111/jvh.13597

[27] Yang H, Wu J, Yuan Y, Huang W, Jia B. Retrospectively seroprevalence study on anti-HEV-IgG antibody in patients with chronic hepatitis or liver cirrhosis in a Chinese teaching hospital. J Med Virol 2019; 91:437–43. 30307619 10.1002/jmv.25335

[28] Amougou Atsama M, Atangana PJA, Noah Noah D, Moundipa PF, Pineau P, Njouom R. Hepatitis E virus infection as a promoting factor for hepatocellular carcinoma in Cameroon: preliminary observations. Int J Infect Dis 2017; 64:4–8. 28847760 10.1016/j.ijid.2017.08.010

[29] Bai MJ, Zhou N, Dong W, Li GX, Cong W, Zhu XQ. Seroprevalence and risk factors of hepatitis E virus infection in cancer patients in eastern China. Int J Infect Dis 2018; 71:42–7. 29656134 10.1016/j.ijid.2018.04.003

[30] Mrzljak A, Dinjar-Kujundzic P, Jemersic L, Vilibic-Cavlek T. The burden of hepatitis e infection in chronic liver diseases in Croatia. Vector Borne Zoonotic Dis 2021; 21:67–8. 32877305 10.1089/vbz.2020.2676

[31] Schulz M, Beha D, Plehm K, Zöllner C, Hofmann J, Schott E. High prevalence of anti-hepatitis e virus antibodies in outpatients with chronic liver disease in a university medical center in Germany. Eur J Gastroenterol Hepatol 2016; 28:1431–6. 27552296 10.1097/MEG.0000000000000729

[32] Covarrubias N, Naveas P, Miranda J, et al Seroprevalencia de virus hepatitis E en donantes de sangre en un hospital universitario en Chile. Revista Chilena de Infectología 2018; 35:455–7. 30534936 10.4067/s0716-10182018000400455

[33] Jaramillo AD, Restrepo L, Mantilla-Rojas C, et al Frequency of antibodies to hepatitis E in blood donors in the municipality of yarumal, Antioquia. Rev Col Gastroenterol 2016; 31:229–34.

[34] Abanto J, Sanchez Boluarte AN, Castillo Y, et al Increased prevalence of antibodies to hepatitis E virus in patients with neurocysticercosis. Am J Trop Med Hyg 2024; 110:1210–3. 38653231 10.4269/ajtmh.23-0856 PMC11154044

[35] Pisano MB, Campbell C, Anugwom C, Ré VE, Debes JD. Hepatitis E virus infection in the United States: seroprevalence, risk factors and the influence of immunological assays. PLoS One 2022; 17:e0272809. 35930611 10.1371/journal.pone.0272809 PMC9355204

[36] Zhao H, Ye W, Yu X, et al Hepatitis E virus superinfection impairs long-term outcome in hospitalized patients with hepatitis B virus-related decompensated liver cirrhosis. Ann Hepatol 2023; 28:100878. 36417965 10.1016/j.aohep.2022.100878

[37] Choi JW, Son HJ, Lee SS, et al Acute hepatitis E virus superinfection increases mortality in patients with cirrhosis. BMC Infect Dis 2022; 22:62. 35042464 10.1186/s12879-022-07050-w PMC8767750

[38] Paternostro R, Traussnigg S, Staufer K, et al Prevalence of anti-Hepatitis E antibodies and impact on disease severity in non-alcoholic fatty liver disease. Hepatol Res 2021; 51:69–79. 33037853 10.1111/hepr.13581

[39] Wang Y, Liu H, Jiang Y, Pan Q, Zhao J. Poor outcomes of acute hepatitis e in patients with cirrhotic liver diseases regardless of etiology. Open Forum Infect Dis 2020; 7:1–5. 10.1093/ofid/ofaa107 PMC7186119 32355864

[40] Akyüz F, Çavuş B, Pınarbaşı B, et al Cryptogenic liver cirrhosis and hepatitis E virus (HEV): are they related? Ann Hepatol 2019; 18:585–9. 31130469 10.1016/j.aohep.2019.01.007

[41] Parfieniuk-Kowerda A, Jaroszewicz J, Łapiński TW, et al High prevalence of anti-HEV antibodies among patients with immunosuppression and hepatic disorders in eastern Poland. Arch Med Sci 2021; 17:675–81. 34025837 10.5114/aoms.2018.79958 PMC8130492

[42] Kumar Acharya S, Kumar Sharma P, Singh R, et al Hepatitis E virus (HEV) infection in patients with cirrhosis is associated with rapid decompensation and death. J Hepatol 2007; 46:387–94. 17125878 10.1016/j.jhep.2006.09.016

[43] McGettigan B, Hernandez-Tejero M, Malhi H, Shah V. Immune dysfunction and infection risk in advanced liver disease. Gastroenterology 2025; 168:1085–100. 39927926 10.1053/j.gastro.2024.08.046 PMC12536737

[44] EASL . EASL clinical practice guidelines on hepatitis E virus infection. J Hepatol 2018; 68:1256–71. 29609832 10.1016/j.jhep.2018.03.005

[45] Haim-Boukobza S, Coilly A, Sebagh M, et al Hepatitis E infection in patients with severe acute alcoholic hepatitis. Liver International 2015; 35:870–5. 24904954 10.1111/liv.12610

[46] Xu HQ, Wang CG, Zhou Q, Gao YH. Effects of alcohol consumption on viral hepatitis B and C. World J Clin Cases 2021; 9:10052–63. 34904075 10.12998/wjcc.v9.i33.10052 PMC8638036

[47] Bala S, Marcos M, Gattu A, Catalano D, Szabo G. Acute binge drinking increases serum endotoxin and bacterial DNA levels in healthy individuals. PLoS One 2014; 9:8–12. 10.1371/journal.pone.0096864 PMC4020790 24828436

[48] Paul RC, Nazneen A, Banik KC, et al Hepatitis E as a cause of adult hospitalization in Bangladesh: results from an acute jaundice surveillance study in six tertiary hospitals, 2014–2017. PLoS Negl Trop Dis 2020; 14:e0007586. 31961861 10.1371/journal.pntd.0007586 PMC6994197

[49] Fantilli AC, Masachessi G, Di Cola G, et al Integrated hepatitis e virus monitoring in central Argentina: a six-year analysis of clinical surveillance and wastewater-based epidemiology. Water Res 2024; 261:122004. 38991242 10.1016/j.watres.2024.122004

[50] Di Cola G, Di Cola G, Fantilli A, et al High circulation of hepatitis E virus (HEV) in pigs from the central region of Argentina without evidence of virus occurrence in pork meat and derived products. Res Vet Sci 2023; 164:105000. 37708830 10.1016/j.rvsc.2023.105000

