# Supplementary material for: Hepatitis E Virus Infection in Patients With Chronic Liver Diseases: A Latin American Multicenter Study
Source: J Infect Dis. 2026 Jan 28;233(4):e1046–55. doi: 10.1093/infdis/jiaf615 (PMC13127749; doi:10.1093/infdis/jiaf615)
Supplement: jiaf615_Supplementary_Data [file jiaf615_supplementary_data.zip › Supplementary_Table_1.docx]

**Supplementary Table 1. Main characteristics of patients stratified by countries**

| **Cohort** | **N** | **Age (median, IQR)** | **Sex (n, % male)** | **Anti-HEV IgG+**  **(n, %) [95% CI]** |
| --- | --- | --- | --- | --- |
| **Argentina** | 283 | 63.0, 14.0 | 151/283, 53.3 | 12/283, 4.2 [2.7-8.2] |
| CLD | 224 | 63.0, 14.0 | 133/224, 59.4 | 10/224, 4.5 [2.3-8.3] |
| HC | 59 | 63.0, 18.5 | 18/59, 30.5 | 2/59, 3.4 [0.4-11.7] |
| **Brazil** | 93 | 63.0, 10.0 | 42/93, 45.2 | 18/93, 19.4 [11.9-28.8] |
| CLD | 69 | 63.0, 8.0 | 41/69, 59.4 | 15/69, 21.7 [12.7-33.3] |
| HC | 24 | 64.0, 12.5 | 1/24, 4.2 | 3/24, 12.5 [2.7-32.4] |
| **Chile** | 162 | 65.0, 11.0 | 70/162, 43.2 | 73/162, 45.1 [37.2-53.1] |
| CLD | 115 | 64.0, 11.0 | 62/115, 53.9 | 57/115, 49.6 [40.1-59.0] |
| HC | 47 | 68.0, 12.3 | 8/47, 17.0 | 16/47, 34.0 [20.9-49.3] |
| **Colombia** | 316 | 63.0, 14.2 | 130/316, 41.1 | 24/316, 7.6 [4.9-11.1] |
| CLD | 259 | 65.0, 12.0 | 124/259, 47.9 | 18/259, 6.9 [4.2-10.8] |
| HC | 57 | 44.0, 29.0 | 6/57, 10.5 | 6/57, 10.5 [4.0-21.5] |
| **Ecuador** | 61 | 63.0, 13.0 | 32/61, 52.4 | 10/61, 16.4 [8.1-28.1] |
| CLD | 61 | 63.0, 13.0 | 32/61, 52.4 | 10/61, 16.4 [8.1-28.1] |
| **Peru** | 56 | 61.0, 10.5 | 33/56, 58.9 | 11/56, 19.6 [10.2-32.4] |
| CLD | 56 | 61.0, 10.5 | 33/56, 58.9 | 11/56, 19.6 [10.2-32.4] |

Abbreviations: CLD = Chronic Liver Disease; HC = Healthy control; 95% CI = 95% confidence interval for proportions; IQR = interquartile range.
