# Supplementary material for: Hepatitis E Virus Infection in Patients With Chronic Liver Diseases: A Latin American Multicenter Study
Source: J Infect Dis. 2026 Jan 28;233(4):e1046–55. doi: 10.1093/infdis/jiaf615 (PMC13127749; doi:10.1093/infdis/jiaf615)
Supplement: jiaf615_Supplementary_Data [file jiaf615_supplementary_data.zip › Supplementary_Table_2.docx]

**Supplementary Table 2. CLD cohort composition**

|  | **Clinical condition** | | | |
| --- | --- | --- | --- | --- |
| **Etiology** | Only cirrhosis  (n=434) | Only HCC  (n=22) | HCC and cirrhosis  (n=193) | Without cirrhosis or HCC  (n=135) |
| ALD | 89 | 3 | 64 | 1 |
| MASLD | 216 | 9 | 71 | 110 |
| Viral hepatitis (chronic Hepatitis B and/or Hepatitis C) | 64 | 5 | 49 | 25 |
| Other | 96 | 5 | 27 | 0 |

*Other etiologies included hemochromatosis, primary biliary cholangitis, secondary biliary cirrhosis, autoimmune hepatitis, cryptogenic hepatitis, and MASH (metabolic dysfunction-associated steatohepatitis), among others. Abbreviations: MASLD = Metabolic dysfunction-associated steatotic liver disease; ALD = alcohol-related liver disease; HCC = Hepatocellular carcinoma.
