# Supplementary material for: Hepatitis E Virus Infection in Patients With Chronic Liver Diseases: A Latin American Multicenter Study
Source: J Infect Dis. 2026 Jan 28;233(4):e1046–55. doi: 10.1093/infdis/jiaf615 (PMC13127749; doi:10.1093/infdis/jiaf615)
Supplement: jiaf615_Supplementary_Data [file jiaf615_supplementary_data.zip › Supplementary_Table_3.docx]

**Supplementary Table 3. Generalized Linear Mixed Model 1 (GLMM)**

**Supplementary Table 3.1. Analysis of deviance***.* Analysis of deviance of the binomial GLMM for the effect of sex, age, and categorical variable 1 [patients with cirrhosis (CR), without cirrhosis (non-CR), Healthy controls (HC)] on anti-HEV IgG seroprevalence.

| **Variable** | **AIC** | **LRT X^2^** | **P-value** ^α^ |
| --- | --- | --- | --- |
| Age | 712.15 | 0.81 | 0.368 |
| Sex | 712.96 | 1.62 | 0.202 |
| Cat-1 | 717.88 | 8.54 | 0.013* |

^α^ P-values were obtained from LRT tests applied within binomial GLMMs with logit link functions. P-values <0.05 were considered significant. Abbreviations: AIC = Akaike information criterion; LRT = Likelihood Ratio Test; Cat-1= Categorical Variable 1

**Supplementary Table 3.2.** **Multiple pairwise comparisons*.*** Multiple pairwise comparisons using Holm method for p-value adjustments.

| **Comparison** | **Estimate** | **Standard Error** | **P-value** ^α^ |
| --- | --- | --- | --- |
| HC vs. CR | -0.303 | 0.275 | 0.269 |
| HC vs. Non | 0.819 | 0.498 | 0.199 |
| CR vs. Non-CR | 1.122 | 0.444 | 0.034* |

^α^ P-values were obtained using Holm method for p-value adjustments. P-values <0.05 were considered significant. Abbreviations: CR = patients with cirrhosis; Non-CR = patients without cirrhosis; HC = healthy controls.
