# Supplementary material for: Hepatitis E Virus Infection in Patients With Chronic Liver Diseases: A Latin American Multicenter Study
Source: J Infect Dis. 2026 Jan 28;233(4):e1046–55. doi: 10.1093/infdis/jiaf615 (PMC13127749; doi:10.1093/infdis/jiaf615)
Supplement: jiaf615_Supplementary_Data [file jiaf615_supplementary_data.zip › Supplementary_Table_4.docx]

**Supplementary Table 4. Generalized Linear Mixed Model 2 (GLMM)**

**Supplementary Table 4.1. Analysis of deviance.** Analysis of deviance of the binomial GLMM for the effect of sex, age, and categorical variable 2 [patients with cirrhosis and alcohol-related liver disease (CR/ALD); with cirrhosis and without alcoholic liver disease (CR/Not-ALD); without cirrhosis or alcoholic liver disease (Not-CR/Not-ALD); healthy controls (HC)] on anti-HEV IgG seroprevalences.

| **Variable** | **AIC** | **LRT X^2^** | **P-value** ^α^ |
| --- | --- | --- | --- |
| Age | 708.53 | 0.66 | 0.415 |
| Sex | 708.06 | 0.19 | 0.662 |
| Cat-2 | 717.05 | 13.18 | 0.004** |

^α^ P-values were obtained from LRT tests applied within binomial GLMMs with logit link functions. P-values <0.05 were considered significant. Abbreviations: AIC = Akaike information criterion; LRT = Likelihood Ratio Test; Cat-2= Categorical Variable 2.

**Supplementary Table 4.2. Multiple pairwise comparisons*.*** Multiple pairwise comparisons using Holm method for p-value adjustments.

| **Comparison** | **Estimate** | **Standard Error** | **P-value** |
| --- | --- | --- | --- |
| HC vs. CR/ALD | -0.894 | 0.377 | 0.089 |
| HC vs. CR/Non-ALD | -0.227 | 0.280 | 0.418 |
| HC vs. Non-CR/Non-ALD | 0.721 | 0.501 | 0.301 |
| CR/ALD vs. CR/Non-ALD | 0.667 | 0.290 | 0.089 |
| CR/ALD vs. Non-CR/Non-ALD | 1.615 | 0.498 | 0.007* |
| CR/Non-ALD vs. Non-CR/Non-ALD | 0.948 | 0.451 | 0.107 |

^α^ P-values were obtained using Holm method for p-value adjustments. P-values <0.05 were considered significant. Abbreviations: CR = patients with cirrhosis; ALD = alcohol-related liver disease; CR/ALD = patients with cirrhosis and alcohol-related liver disease; CR/Non-ALD = patients with cirrhosis and without ALD; Non-CR/Non-ALD = patients without cirrhosis or ALD; HC = healthy controls.
