# Supplementary material for: Hepatitis E Virus Infection in Patients With Chronic Liver Diseases: A Latin American Multicenter Study
Source: J Infect Dis. 2026 Jan 28;233(4):e1046–55. doi: 10.1093/infdis/jiaf615 (PMC13127749; doi:10.1093/infdis/jiaf615)
Supplement: jiaf615_Supplementary_Data [file jiaf615_supplementary_data.zip › Supplementary_Table_5.docx]

**Supplementary Table 5. Generalized Linear Mixed Model 4 (GLMM)**

**Supplementary Table 5.1. Analysis of deviance*.*** Analysis of deviance of the binomial GLMM for the effect of sex, age, and categorical variable 3 [patients with alcohol-related liver disease (ALD); without alcohol-related liver disease (Non-ALD); healthy controls (HC)] on anti-HEV IgG seroprevalences.

| **Variable** | **AIC** | **LRT X^2^** | **P-value** ^α^ |
| --- | --- | --- | --- |
| Age | 713.62 | 1.16 | 0.281 |
| Sex | 712.72 | 0.26 | 0.606 |
| Cat-3 | 717.88 | 7.42 | 0.024* |

^α^ P-values were obtained from LRT tests applied within binomial GLMMs with logit link functions. P-values <0.05 were considered significant. Abbreviations: AIC = Akaike information criterion; LRT = Likelihood Ratio Test; Cat-3= Categorical Variable 3.

**Supplementary Table 5.2. Multiple pairwise comparisons*.*** Multiple pairwise comparisons using Holm method for p-value adjustments.

| **Comparison** | **Estimate** | **Standard Error** | **P-value** |
| --- | --- | --- | --- |
| HC vs. ALD | -0.898 | 0.380 | 0.036* |
| HC vs. Non-ALD | -0.133 | 0.281 | 0.635 |
| ALD vs. Non-ALD | 0.765 | 0.286 | 0.022* |

^α^ P-values were obtained using Holm method for p-value adjustments. P-values <0.05 were considered significant. Abbreviations: ALD = patients with alcohol-related liver disease; Non-ALD = patients without alcohol-related liver disease; HC = Healthy controls.
